# Synthesis, Characterization, and Evaluation of the Adsorption Behavior of Cellulose-Graft-Poly(Acrylonitrile-co-Acrylic Acid) and Cellulose-Graft-Poly(Acrylonitrile-co-Styrene) towards Ni(II) and Cu(II) Heavy Metals

**DOI:** 10.3390/polym16030445

**Published:** 2024-02-05

**Authors:** Amany S. El-Khouly, Yoshiaki Takahashi

**Affiliations:** 1 Department of Chemistry, College of Science, King Faisal University, Al Ahsa 31982, Saudi Arabia; 2 Department of Chemistry, Faculty of Science, Tanta University, Tanta 31527, Egypt; 3 Division of Advanced Device Materials, Institute for Materials Chemistry and Engineering, Kyushu University, Kasuga 816-8580, Japan; ytak@mm.kyushu-u.ac.jp

**Keywords:** modified cellulose, graft copolymerization, monomer mixture, metal ion adsorption, optimization

## Abstract

In this study, the synthesis and characterization of grafted cellulose fiber with binary monomers mixture obtained using a KMnO_4_/citric acid redox initiator were investigated. Acrylonitrile (AN) was graft copolymerized with acrylic acid (AA) and styrene (Sty) at different monomer ratios with evaluating percent graft yield (GY%). Cell-g-P(AN-co-AA) and Cell-g-P(AN-co-Sty) were characterized by SEM, FT-IR, ^13^C CP MAS NMR, TGA, and XRD. An AN monomer was used as principle-acceptor monomer, and GY% increases with AN ratio up to 60% of total monomers mixture volume. The adsorption behaviors of Cell-g-P(AN-co-AA) and Cell-g-P(AN-co-Sty) were studied for the adsorption of Ni(II) and Cu(II) metal ions from aqueous solution. Optimal adsorption conditions were determined, including 8 h contact time, temperature of 30 °C, and pH 5.5. Cell-g-P(AN-co-AA) showed maximum adsorption capacity of 435.07 mg/g and 375.48 mg/g for Ni(II) and Cu(II), respectively, whereas Cell-g-P(AN-co-Sty) showed a maximum adsorption capacity of 379.2 mg/g and 349.68 mg/g for Ni(II) and Cu(II), respectively. Additionally, adsorption equilibrium isotherms were studied, and the results were consistent with the Langmuir model. The Langmuir model’s high determinant coefficient (R^2^) predicted monolayer sorption of metal ions. Consequently, Cell-g-P(AN-co-AA) and Cell-g-P(AN-co-Sty) prepared by a KMnO_4_/citric acid initiator were found to be efficient adsorbents for heavy metals from wastewater as an affordable and adequate alternative.

## 1. Introduction

Cellulose remains the most important raw material used in the textile industry, which can be extracted from cotton, wood, and plant- based cellulosic fibers. Its physical and chemical properties have been modified by different chemical modifications, cross-linking [1,2,3,4], substitution [5], functionalization, and by grafting with different types of monomers [6,7,8,9,10,11,12,13,14]. Cellulose and its modified fibers are used in various fields, such as paper, biomedical, biological, packaging, composite preparation, and adsorption. In the last two decades, a remarkable amount of global attention has been paid to the usage of cellulose-based sorbent in the treatment and removal of toxic heavy metals as pollutants from the wastewater.

There are many toxic and harmful organic and inorganic contaminants on the surface and water resources. Heavy metal ion pollution has increased significantly in our society due to the accelerated pace of industrialization and other human inputs [15,16]. The existence of heavy metals in drinkable water at levels far over the allowable limit, in particular, is gravely affecting human health and the environment, having long-term consequences on the human body [17,18,19,20], mainly due to their no or low biocompatibility and high toxicity [21]. Water purification procedures, such as ion exchange, sedimentation, coagulation, ultrafiltration, adsorption, absorption, and others have been reported in the literature [22,23]. Among these, adsorption is considered the most economic, available, and effective technique for the removal of heavy metals from water. It has several characteristics, including effectiveness, low cost, higher tendency, and reusability to remove contamination [24]. Hence, it is required to develop an efficient biobased substance to remove heavy metals from water. In comparison to inorganic adsorbents, organic compounds generated or developed from natural biopolymers such as starch [25,26], chitosan [27,28,29,30], cellulose [31,32], pectin [33,34], and others are more appropriate due to their sustainability and biodegradability.

In recent decades, cellulose and its bio-renewable fiber as adsorbents have become the most common low-cost biopolymer. Since the adsorption capacity of cellulosic raw materials is restricted, cellulose needs to be chemically treated to produce an effective adsorption activity [35,36,37]. The coveted recyclable, non-toxic, biodegradable, and environmentally beneficial features of cellulose-based grafted copolymers have always piqued the interest of researchers. The characteristics and chemical structures of the monomers affect the grafted copolymers’ hydrophilic/hydrophobic and sorption efficiencies in addition to their thermal and chemical stabilities. Grafted cellulose with methacrylamide (MAm) in presence of N,N-methylene bisacrylamide were prepared and applied in sorption of Fe(II), Cu(II) and Cr(VI) ions [38]. Adsorption properties of the synthesized thermoresponsive cellulose-graft-poly(N-isopropyl acrylamide) copolymer for Cu(II), Pb(II), Ni(II) and Cd(II) ions have been studied [39]. Graft copolymerization of glycidyl methacrylate onto cellulose was carried out by γ-initiated graft polymerization and was applied as an adsorbent for Pb(II), Cu(II), and Cd(II) ions [40]. In addition, it was also prepared by microwave-assisted technique for the adsorption of Hg(II) [41]. For the sorption of Co(II) ions, cellulosic cotton fibers, modified with methacrylic acid and glycidyl methacrylate, were synthesized utilizing gamma irradiation [42]. Additionally, graft copolymerization of cellulose cotton fibers with polyacrylonitrile was prepared in order to extract and eliminate Au(III), Pd(II), and Ag(I) precious metal ions [43]. An adsorbent for Cr(VI) ions from water was developed by grafting acrylonitrile (AN) onto recovered cellulose from sisal fiber [44] as well as grafted acrylic acid (AA) to adsorb Ni(II) and Cu(II) metal ions [45]. Cellulosic materials extracted from Gosweilerodenron balsamiferum wood residues were graft-copolymerized with AN and AA and then examined to eliminate Cu(II) and Cd(II) from wastewater medium [46]. Cellulose Nano Crystals (CNCs) extracted from Banana fiber were grafted with butyl acrylate (BA) using ceric ammonium nitrate (CAN) as an initiator and its adsorption efficiency was studied for the removal of Pb(II) ions from the aqueous [47]. To remove Cu^2+^ from aqueous solutions, bamboo cellulose nanofibers-graft-poly (acrylic acid) (BCN-g-PAA) and bamboo cellulose nanofibers graft-poly (acrylic acid)/sodium humate (BCN-g-PAA/SH) were prepared and used as biosorbents [48]. 2-mercaptobenzamide modified itaconic acid-grafted-magnetite nanocellulose composite [P(MB-IA)-g-MNCC] was prepared using EGDMA as a cross linking agent and K_2_S_2_O_8_ (KPS) as a free radical initiator for adsorbing Hg(II) metal ions from aqueous solutions [49]. Nanofabricated cellulose-graft-(2-hydroxyethyl methacrylate) (HEMA/CNF) was firstly synthesized using the microwave-assisted technique in the presence of ceric ammonium nitrate (CAN) initiator, then was fabricated by electrospinning using N,N-dimethylacetamide-LiCl solvent for adsorption of Cd(II) and Pb(II) metal ions from waste water [50]. Acrylamide and AN mixture was grafted onto cellulose by using ascorbic acid and hydrogen peroxide as redox initiator for removal the toxic Cd(II), Pb(II), and Zn(II)metal ions from the water [51]. For the purpose of studying the sorption behavior for Fe(II), Cu(II), and Cr(VI) ions, cellulose grafted with 2-hydroxy methacrylate and its binary monomer mixture with AA, AN, and acrylamide were examined [52]. Cellulose was first extracted from pine needles, then grafted by glycidyl methacrylate with other binary monomers to adsorb Fe(II), CU(II), and Cr(VI) ions [53]. 2-Acrylamido-2-methpropane sulfonic acid and its binary mixture with acrylonitrile were grafted onto cellulose extracted from rice husk for studying their adsorption efficiencies towards Pb(II) metal ions [54]. Extracted cellulose from agricultural residue rice husk was grafted with N-isopropylacrylamide (NIPAM) and comonomer acrylic acid (AAc) using potassium persulfate as free radical initiator and examined as adsorbents for Cu(II), Ni(II), and Pb(II) metal ions from wast water [55]. Also, Cellulose extracted from agro-waste rice Cell-g-NIPAM-co-GMA graft copolymer was prepared by KPS and its adsorption behavior was examined towards Pd(II), Ni(II), and Cu(II) removal from aqueous solution [56]. Cell-g-HEMA-co-GMA graft copolymer prepared by KPS initiator was used as adsorbent for the removal of different divalent metal ions [57].

The purpose of our study was to prepare an improved chemically modified cotton cellulose fiber (Cell) that exhibited higher thermal stability and efficient adsorption capacity using low-cost and simple method, KMnO_4_/citric acid redox initiator [58], by introducing new binary Acrylonitrile (AN)/acrylic (AA) acid and also acrylonitrile/styrene (Sty) mixtures into cellulose fibers as novel graft copolymers, compared to individual monomers as well as to their other binary mixtures [32,35,37,55,59]. Moreover, we aimed to assess the behavior of Cell-g-P(AN-co-AA) and Cell-g-P(AN-co-Sty) for Ni(II) and Cu(II) metal ions and to determine the underlying mechanisms. Accordingly, the adsorption behavior of Cell-g-P(AN-co-AA) and Cell-g-P(AN-co-Sty) towards Ni(II) and Cu(II) metal ions from aqueous medium were examined at different adsorption factors, such as adsorption contact time, pH medium, adsorption temperature, and initial metal ion concentration, to determine the optimized adsorption conditions. The adsorption data were applied in different adsorption isotherm models for evaluating the sorption mechanism and also were compared with other studies [32,35,37,55,56,57,59] to investigate the effect of the graft polymerization initiation system and the type of binary monomer mixture on the adsorption efficiencies towards heavy metal ions removal.

## 2. Materials and Methods

### 2.1. Materials

Cellulose cotton fiber (75 g/m^2^), produced by the El-Mahalla Company for Spinning and Weaving—EL-Mahalla ELkobra, Egypt, was used. Acrylonitrile (AN), acrylic acid (AA), and styrene (Sty) were received from Wako Pure Chemical Industries, Ltd., Tokyo, Japan, and were purified by distillation. Potassium permanganate, dimethylformamide (DMF), and ethyl acetate were purchased from Kishida Chemical Co., Ltd., Osaka, Japan and used as received. Citric acid, acetone, methanol, sodium hydroxide, nickel chloride, and copper chloride were purchased from Wako Pure Chemical Industries, Ltd., Tokyo, Japan, and were used without further purification.

### 2.2. Graft Copolymerization of Binary Vinyl Monomers Mixture onto Cellulose Fibers

Grafting of the binary mixture was carried out using the most efficient conditions obtained from our previous work [58]. Briefly, using a cellulose material to liquid ratio of 1:100 (wt/vol), one gram of pure cellulose was impregnated in 0.05 mol/L aqueous KMnO_4_ solution. The treatment method was run for 30 min at 60 °C under continuous shaking, then the samples were washed several times using distilled water. Subsequently, the optimized conditions of the graft copolymerization obtained from our previous work [58] were used for grafting of AN-other monomer mixtures (AA or Sty) onto cellulose at various volume (%) ratios: 10:0; 8:2; 6:4; 4:6; 2:8; and 0:10 to attain a total monomer ratio of 10 vol% in the grafting solution. After the reaction time (one hour), the sample was removed from the flask and washed with dist. H_2_O several times to remove the unreacted monomers. The homopolymer was removed from the grafted samples by washing with DMF for AN homopolymer, ethyl acetate for AA homopolymer, and methanol for Sty homopolymer. Finally, the pure grafted cellulose samples were dried at 40 °C for 24 h and the percent graft yield (GY%) was calculated using the following equation.

(1) $$GY\;\left(\%\right)=\frac{W_{2}-W_{1}}{W_{1}}\times 10$$

where *W*_1_ and *W*_2_ are the weights of initial cellulose, and grafted cellulose, respectively.

### 2.3. Characterization

#### 2.3.1. Scanning Electron Microscopy (SEM)

Surface morphology of the pure cellulose and grafted samples were examined by Hitachi S-4100 SEM (Hitachi High-Tech, Tokyo, Japan) at an accelerating voltage of 10 KV.

#### 2.3.2. Solid State NMR Measurement (^13^C CP MAC NMR)

Solid state ^13^C NMR analysis was measured on Delta2-NMR spectrometer (B0 = 9.4 T) (Jeol Ltd., Tokyo, Japan) with resonance frequency of 100.52 MHz at Kyushu University. Samples were held in Aurum tube-capped zirconia rotors (6 mmϕ). The decoupling with TPPM at γB1/2P = 100 kHz and the 5-KHz spinning speed were used. Take note that through a comparison of the data for untreated cellulose, the compatibility of the data using both different instruments/conditions were proven.

#### 2.3.3. Fourier Transform Infrared Spectroscopy (FT-IR Spectra)

The Bio-Rad FTS 6000 spectrometer (Tokyo, Japan) was used to record IR spectra for cellulose and its graft copolymer samples with 32 scans at a maximum resolution of 2 cm^−1^. A 10 μm-thick layer of material was used by weighing 1.0 mg of sample pressed onto to potassium bromide. The scanned range of FT-IR spectra was 400–4000 cm^−1^.

#### 2.3.4. Thermogravimetric Analysis (TGA)

A pure cellulose, cellulose graft copolymer with AN and with binary vinyl monomers were characterized by TGA by using Seiko TG/DTA6300 (Seiko Instruments Inc., Chiba, Japan) in nitrogen atmosphere. The weight of measured samples were in the range of 1.1–1.4 mg and TGA were recorded from 28 to 530 °C and heated at a rate of 10 °C/min.

#### 2.3.5. X-ray Measurements

The X-ray diffractogram (XRD) of cellulose samples were measured with Rigaku RINT2100H/KLC X-ray diffraction instrument (Rigaku, Tokyo, Japan) with Ni-filtered Cu Kα radiation at room temperature with 2θ range of 5–30°.

### 2.4. Adsorption of Metal Ions from Aqueous Medium

In distilled water, 50 mL solutions of each metal ion were prepared by dissolving correctly weighed metal chlorides. Each batch experiment used 0.05 g of cellulose samples in metal ion solution. The instrument UV-Vis spectroscopy (Hitachi U-3200 spectrophotometer, Hitachi High-Tech, Tokyo, Japan) was used to determine the concentration of metal ions remaining in the solution after a predetermined duration. Influence of the medium pH (2.0–6.5), contact time (0.5–36 h), temperature (20–45 °C), and concentration of metal ion (20–1000 mg/L) on the adsorption efficiency of the grafted cellulose samples was also investigated in batch tests. The following Equations (2) and (3) were used to determine the adsorption capacity ‘q_e_’ and the percent uptake (Pu) [60,61].

(2) $$q_{e}=\frac{C_{i}-C_{e}}{Wt}V$$

(3) $$Pu=\frac{C_{i}-C_{e}}{C_{i}}\times 100$$

where C_i_ = initial concentration of metal ions (mg/L), and C_e_ = equilibrium concentration in medium after time (mg/L), v = volume of the metal ion solution (L), and Wt = weight of the adsorbent sample (g).

All the graft copolymerization and adsorption experiments were repeated three times. Results were expressed as mean ± SD.

## 3. Results and Discussion

### 3.1. Graft Copolymerization

The individual grafting of acrylic acid (AA), and styrene (Sty) have been studied by using redox system of KMnO_4_/citric acid with the efficient reaction conditions obtained before in our previous work [58]. It was found that the grafting of AA and Sty have no or slight efficiencies onto cellulose in presence of the redox system compared with AN, which has high effect as shown in Table 1. From these results, the grafting of AA and Sty monomers onto cellulose were not active to attach directly to cellulose backbone by KMnO_4_/citric acid redox system. According to the obtained results and our previous studies [58], AN was used as the principle and acceptor monomers to the other monomer in the grafting binary monomers mixtures.

**Figure 1:**
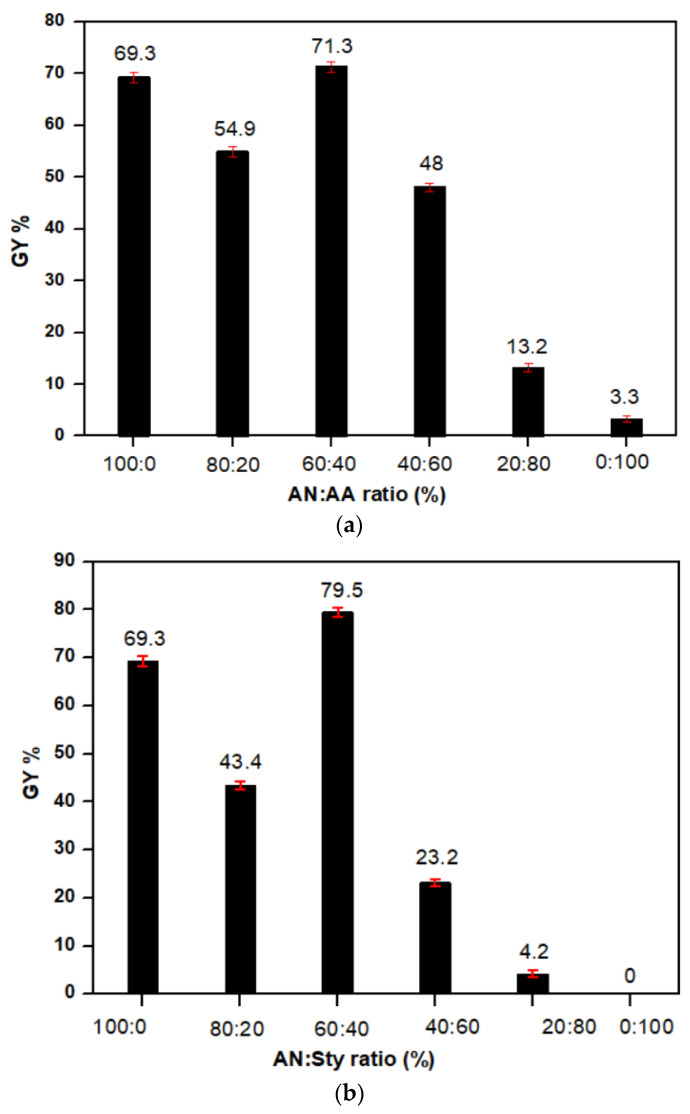
(**a**) Percent graft yield (GY%) of AN + AA binary mixture onto cellulose, monomer concentration = 10 v%, time = 60 min, [KMnO_4_] = 0.05 mol/L, M:L ratio = 1:100, temperature = 70 °C, [citric acid] = 0.02 mol/L. (**b**) Percent graft yield (GY%) of AN + Sty binary mixture onto cellulose, monomer concentration = 10 v%, time = 60 min, [KMnO_4_] = 0.05 mol/L, M:L ratio = 1:100, temperature = 70 °C, [citric acid] = 0.02 mol/L.

The grafting behavior of the binary mixtures of AN with other monomers at the abovementioned reaction conditions are presented in Table 1 and Figure 1a,b. Grafting of binary vinyl monomer mixtures onto cellulose was carried out using AN as the principal monomer [62,63,64]. The higher percentage of grafting in the binary monomer mixtures can be explained by the fact that the insertion of the electron-acceptor monomers, AN, as well as the electron–donor monomers, AA and Sty, to AN increase the reactivity of the monomers towards grafting.

In the case of AN/AA and AN/Sty mixtures, as shown in Figure 1a,b, grafting of AA on the cellulose is very low. However, in the case of the mixture, the percent graft yield (GY%) increases with increasing the ratio of AN up to 60% from the total monomer percentage (10%). This is attributed to the higher reactivity of AN over that of AA and Sty, and thereby leading to less free radical sites on the monomeric units, and hence, a decreased graft yield has been shown by decreasing the percentage of AN. As a result, GY% depends on the monomer ratio in the initial mixture and on their concentration as well as shown in Table 1.

### 3.2. Characterization of the Grafted Cellulose

#### 3.2.1. SEM Analysis

**Figure 2:**
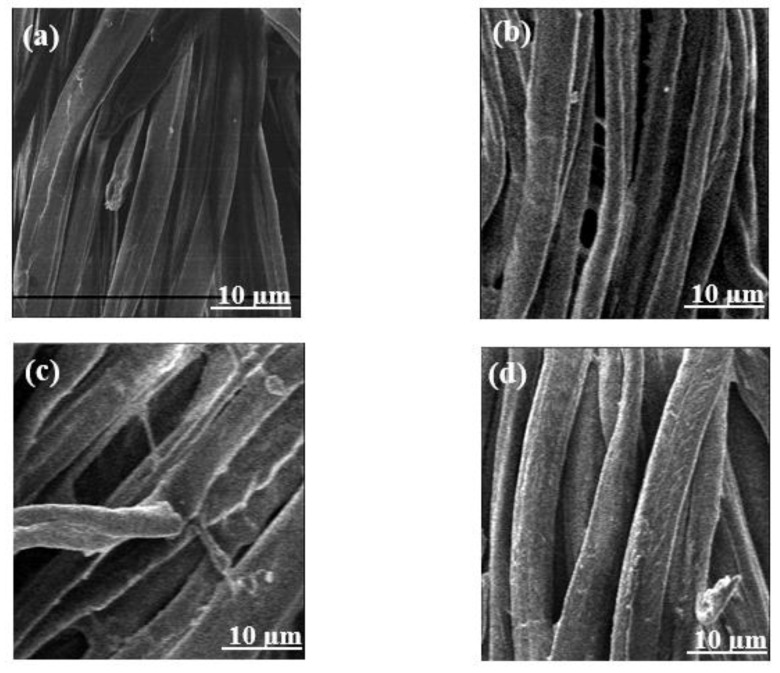
SEM images of (**a**) pure cellulose, (**b**) cell-g-PAN, (**c**) Cell-g-P(AN-co-AA), and (**d**) Cell-g-P(AN-co-Sty).

Structural and morphological properties of pure cellulose (pure-cell), cellulose-graft-polyacrylonitrile (Cell-g-PAN), cellulose-graft-poly(acrylonitrile-co-acrylic acid) (Cell-g-P(AN-co-AA)), and cellulose-graft-poly(acrylonitrile-co-styrene) (Cell-g-p(AN-co-Sty)) were measured through SEM analysis (Figure 2). After graft copolymerization, the cellulose fibers’ smooth surface transformed into a rough and more thick structures with tiny clumps of the grafted polymers attached to it, especially with grafted binary mixture cell-g-P(AN-co-AA) and Cell-g-P(AN-co-Sty).

#### 3.2.2. FT-IR Analysis

**Figure 3:**
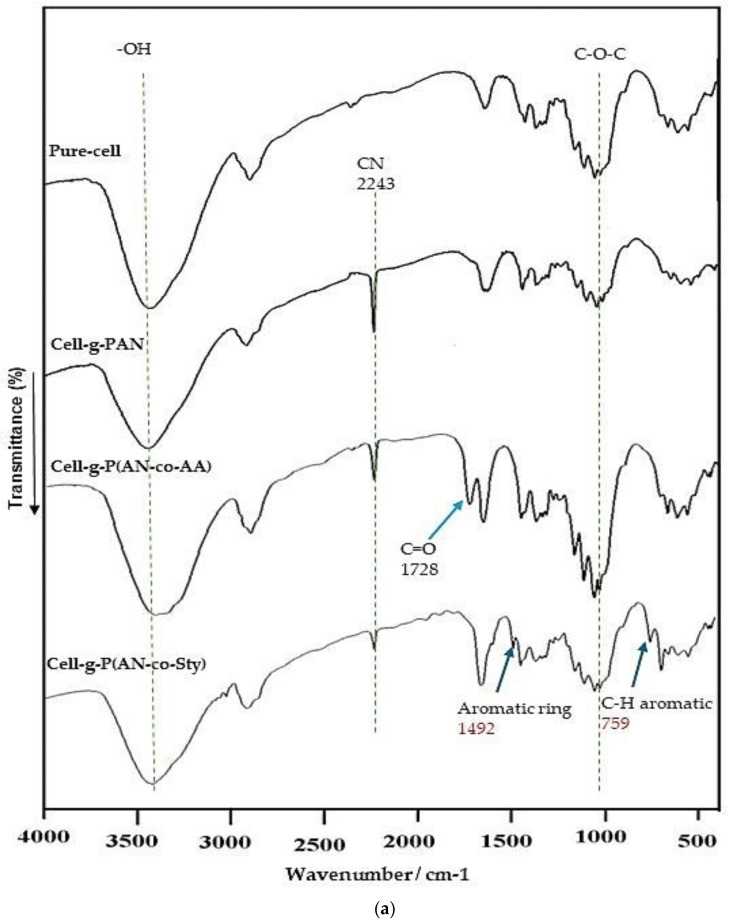

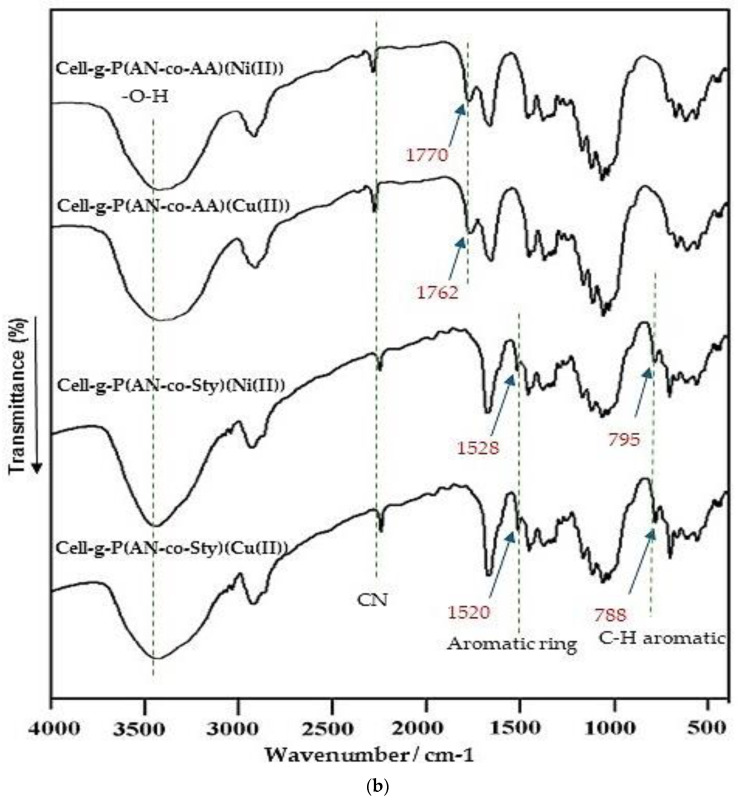
(**a**) FT-IR Spectra of pure cellulose and its grafted samples with monomer mixtures. (**b**) FT-IR of grafted cellulose with monomer mixtures after adsorption of Ni(II) and Cu(II) metal ions.

Figure 3 shows the IR spectra of pure-cell, cell-g-PAN, C-g-P(AN-co-AA), and C-g-P(AN-co-Sty). The FTIR Spectra of all grafted cellulose samples showed the typical characteristic peaks of cellulose. The absorption peaks around 3360 cm^−1^ were related to the hydroxyl groups (–OH) and the band at 2905 cm^−1^ was attributed to C–H stretching vibration. Other bands at 1430 cm^−1^ and 1050 cm^−1^ correspond to the –CH_2_ bending vibration and C–O–C stretching vibration in the glucopyranose ring, respectively. In the IR spectrum of Cell-g-PAN, the band at 2243 cm^−1^ was assigned to the nitrile group (CN). Compared with the IR spectrum of Cell-g-PAN, the IR spectra of cellulose graft poly(acrylonitrile-co-acrylic acid) has shown an additional absorption band at 1728 cm^−1^ due to the carbonyl group (C=O) of acrylic acid (Figure 3). Moreover, another absorption band at 2243 cm^−1^ was observed due to CN group of acrylonitrile in comparison to pure cellulose. The distinctive peaks of cellulose include a strong and broad band at 3500–3000 cm^−1^, which represents the O–H bond from AA. Hence, this supplied evidence for the grafting of both monomers onto cellulose. In the IR spectrum of Cell-g-P(AN-co-Sty), the peak at 889 cm^−1^ was related to C–H aromatic group of Sty, as well as bands at 1492 cm^−1^ and 3150–2850 cm^−1^ range, corresponding to the aromatic ring mode and aromatic C–H/CH_2_ stretching of polystyrene. According to these results, AN, AA, and Sty successfully introduced pure cellulose by the graft-copolymerization using KMnO_4_/citric acid redox system. Figure 3b represents IR of C-g-P(AN-co-AA) and Cell-g-P(AN-co-Sty) after adsorption of Ni(II) and Cu(II) ions. The peak of C=O group in Cell-g-p(AN-co-AA) exhibited low intensity and shifted to 1770 and 1762 cm^−1^, due to the formation of complexes with Ni(II) and Cu(II) metal ions [37,65]. Furthermore, the peaks of C-H aromatic group and aromatic ring in Cell-g-P(AN-co-Sty) shifted to1528 and795 cm^−1^ after adsorption of Ni(II), respectively, and also shifted to 795 and 788 cm^−1^ after adsorption of Cu(II) ions, respectively. In addition, the peaks related to CN slightly shifted to 2462 and 2458 cm^−1^ in Cell-g-P(AN-co-AA) and Cell-g-P(AN-co-Sty), respectively. This strongly supports the coordination between Cell-g-P(AN-co-AA), Cell-g-P(AA-co-Sty), and heavy metal ions, which suggests that carboxylic group and aromatic rings are involved in the adsorption of the heavy metal ions from the aqueous medium.

#### 3.2.3. Solid State NMR Analysis

Cellulose samples grafted with the binary mixtures of vinyl monomers were examined by ^13^C Cp MAS NMR analysis. As previously discussed, graft-cellulose with AN exhibits a spectrum with an intense and clearly recognizable peak at 125 ppm, attributed to the nitrile carbons, and a broad resonance at about 40 ppm, according to the polyacrylonitrile (PAN) backbone’s carbon resonance [58].

**Figure 4:**
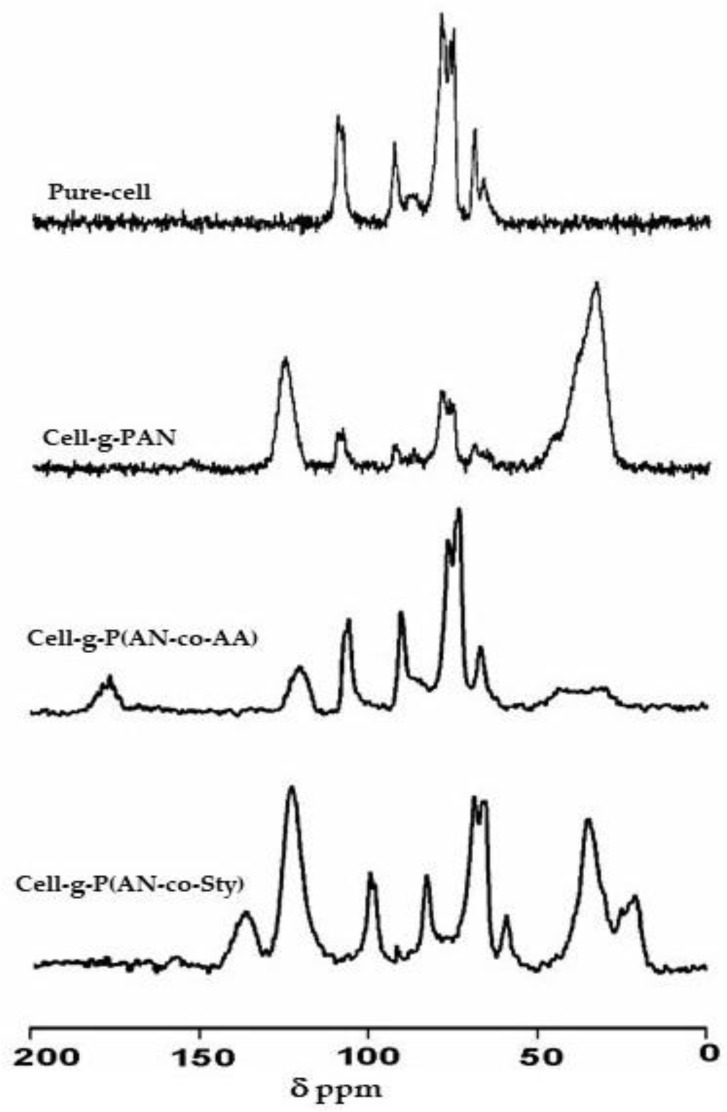
^13^C CP MAS NMR spectra of pure cellulose, cellulose-graft-polyacrylonitrile, and its binary monomer mixtures with AA and Sty.

In Figure 4, the spectrum of the cellulose graft poly(acrylonitrile-co-acrylic acid) is reported. Resonance belonging AA are well observable: the resonance peaks due to AA backbone are found at 31 and 42 ppm which overlap with peak of carbon resonance of PAN backbone and the resonance of carbonyl carbon is detected at 176 ppm. The grafting reaction has no effect on the cellulose backbone structure; in fact, the spectral range 60–116 ppm remains unaltered. Also, the characteristic signals for AN are observed, along with the AN backbone at 40 ppm and nitrile group resonance at 137 ppm.

The ^13^C spectrum of cellulose graft poly(acrylonitrile-co-styrene) shows extra peaks resulting from the polystyrene bonded to the polyacrylonitrile grafts. The main chain CH and CH_2_ carbons give rise to the peaks at 22–28 ppm, while the tertiary aromatic carbons resonate at the clear intense peak of 122 ppm and the quaternary aromatic carbon resonates at approximately 140 ppm.

#### 3.2.4. Thermal Analysis

**Figure 5:**
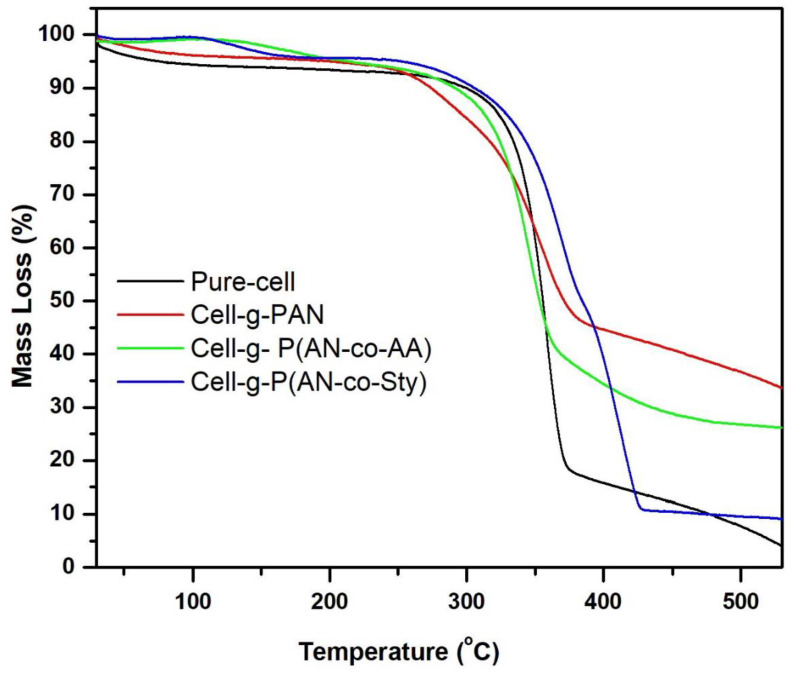
TG graphs of pure cellulose and grafted cellulose with various monomers mixtures.

Thermal characterization and degradation of cellulose and its grafted samples with AN and mixture of monomers were studied by TGA as shown in Table 2 and Figure 5. TGA of pure cellulose showed 6% of weight loss at up to 100 °C, which represents the water desorption stage. In addition, this peak was observed in cell-g-PAN with 4% weight loss. The difference between pure cellulose and cell-g-PAN is attributed to the presence of the hydrophobic PAN chain polymer. In the decomposition stage from 250 °C to 370 °C, there is a difference between TG of cellulose and TG of cell-g-PAN in cellulose. At 250–300 °C, there is about 10% weight loss in the grafted samples which is comparable to thermal decomposition of PAN [58]. In the range 300–370 °C, a sharp weight loss was observed in pure cellulose with 78% and in cell-g-PAN with 36%, which can be referred to depolymerization of cellulose backbone chain through the degradation of glycosidic bonds (C–O–C) with the decomposition of the grafted chain. The final decomposition temperature was observed at 530 °C with 2% residue left in pure cellulose and 33% in cell-g-PAN.

In the case of cell-g-p(AN-co-AA) sample, the weight loss of water desorption stage is lower than cell-g-PAN, which is attributed to more hydrophobic polymers with the grafted binary mixture. Also, the slight increase in the thermal stability in the temperature range of 250–300 °C with 4.2% weight loss was observed compared to the same stage in cell-g-PAN. The weight loss in the temperature range of 300–370 °C was 49.9%. Moreover, there was an extra decomposition stage at the temperature range of 370–465 °C, which is attributed to the decomposition of carboxylic group from AA [29,59,66], with 15.13% weight loss. The residue at 350 °C was 26%. The TGA of grafted copolymer cell-g-P(AN-co-Sty) showed the degradation stage in the temperature range of 260–380 °C with 41% weight loss, which indicated higher thermal stability compared with pure cellulose, cell-g-PAN and cell-g-p(AN-co-AA) as shown in Table 2 and Figure 5. Furthermore, there is an extra stage starting from 390 to 434 °C, which is attributed to the degradation of the Sty polymer chain. The final decomposition temperature was at 530 °C with 9% residue left. From TG curves, it can be observed that grafting of AN decreases the thermal stability of cellulose, while grafting of the binary mixture increases the thermal stability of cellulose, compared to individual monomers as well as to their other binary mixtures [32,35,37,55,59], especially in the presence of polystyrene.

#### 3.2.5. X-ray Diffraction Analysis

**Figure 6:**
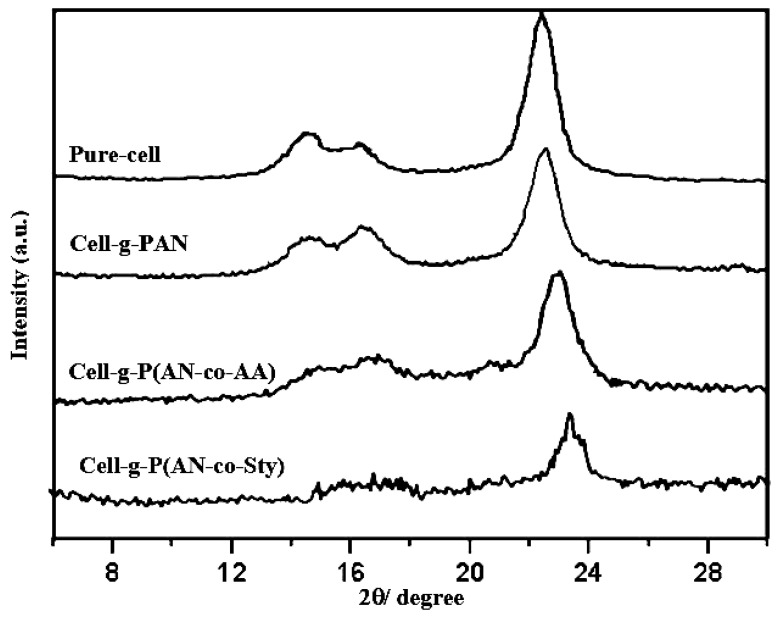
X-ray diffraction of pure cellulose and grafted cellulose with monomer mixture of 60:40 ratio of AN/vinyl monomer.

The diffractograms of grafted cellulose with different monomer mixtures are presented in Figure 6 and Figure S1a–d. X-ray of pure cellulose shows diffraction peaks at diffraction angles (2θ) values of 14.5, 16.3, and 22.5, which corresponds to (101), (10l’) and (002) reflections of cellulose I, respectively [58]. The X-ray curves in all Figures show that the intensities of the cellulosic peaks decrease sharply on increasing the ratio of AN in the grafting process. The values of the crystallinity degree (Cr%) are calculated [58,67] and are listed in Table 3. In the case of grafting using AN/AA, the Cr% is different than that grafting using AN only. Also, cellulose-graft poly(acrylonitrile-co-styrene) samples showed a decreasing in Cr%. This decrease of Cr% may be due to the interference of bulky pendant chains of PSty. and PAN grafted onto cellulose molecules.

In Figure 6 and Figure S1a, we can observe that the ratios of 80:20 and 60:40 of the monomer mixtures have more effect on the crystallinity of cellulosic fiber compared with the other ratios, where the monomer mixture of AN/Sty has observed effect in comparison with grafting of AN onto cellulose with the same volume concentration of the monomer. From the data listed in Table 3, the degree of crystallinity is affected by GY%, and also is affected by the type and the ratio of the binary monomer mixture used in the grafting onto cellulose. Generally, the decline in crystallinity ratio of the grafted cellulose samples is the result of the disorientation of the cellulose crystals upon grafting with different monomers, chiefly with AN/Sty binary monomer mixtures.

### 3.3. Sorption Properties of Cellulose Graft Copolymer

The following preliminary experiments were performed to investigate the optimal conditions for the metal ions adsorption test from the aqueous solution by the grafted cellulose samples.

#### 3.3.1. Effect of the pH Medium and Contact Time

**Figure 7:**
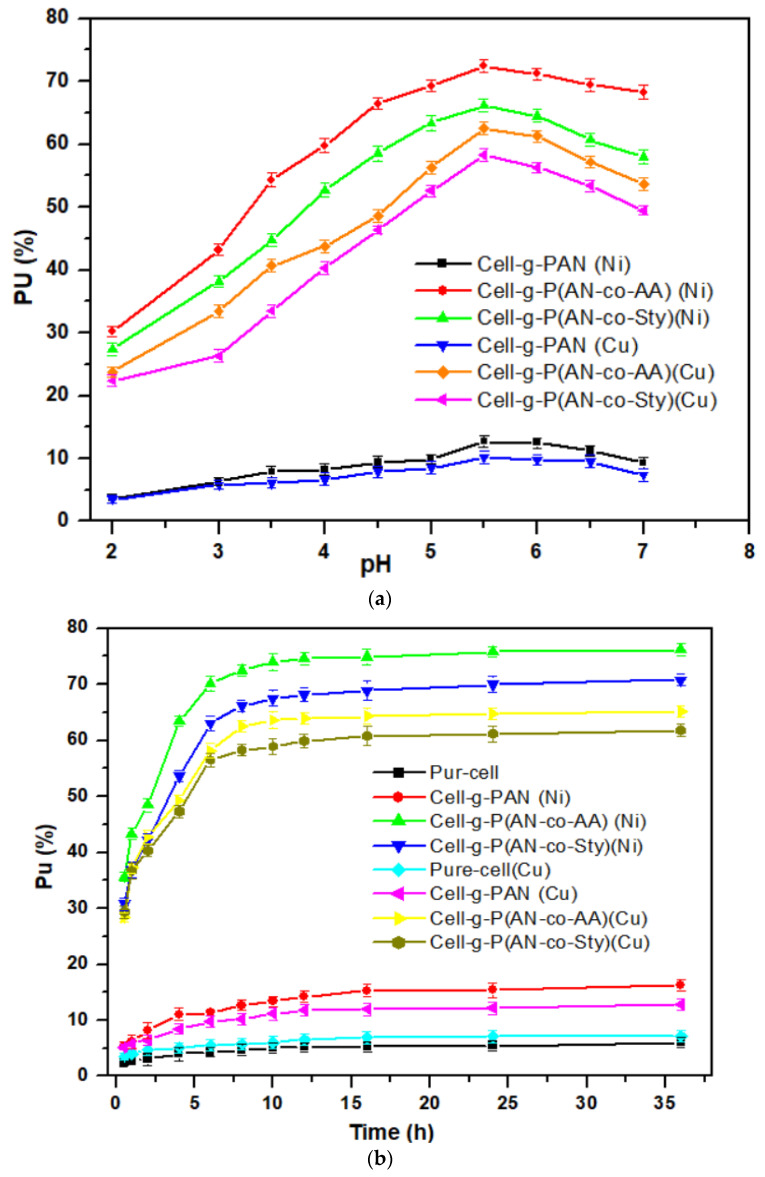
(**a**) Effect of pH on the amount uptake by different adsorbents. (**b**) Effect of contact time on the amount uptake by different adsorbents.

Pure cellulose, Cell-g-P(AN-co-AA), and Cell-g-P(AN-co-Sty) with higher percent graft yield were examined as adsorbents to evaluate their optimum adsorption efficiencies towards Ni(II) and Cu(II) at 30 °C and 8 h. Firstly, the pH of the solution medium was examined in the range of 2.0–6.5 to determine the most suitable one for metal adsorption. The pH value strongly affects metal ions adsorption, precipitation, and charge surface of the adsorbent samples, as shown in Figure 7a. With increasing the pH of the medium, the sorption of Ni(II) and Cu(II) metal ions proportionally increases up to pH 5.5 with cell-g-p(AN-co-AA) and cell-g-P(AN-co-Sty). The low adsorption efficiency of metals ions at a pH lower than 4.0 can be ascribed to the competitive behavior of the adsorption of metal ions and hydronium ions (H_3_O^+^) for the same adsorption sites of the copolymers. Additionally, the metal ions were found as hydroxides when the initial pH was higher than 6.0, which caused turbid solutions and interfered with other parallel studies. As a result, the pH of medium for the other adsorption experimental tests with factors was carried out at pH 5.5.

In Figure 7b, the effect of contact time on the adsorption efficiency of cellulose graft copolymer samples was examined for Ni(II) and Cu(II) metal ions at pretest condition of pH 5.5, 50 mg adsorbent, 600 mg/L metal ion concentration, temperature (30 °C), and 50 mL volume of the adsorption solution. The adsorption behaviors of the metal ions are similar, which is very fast with increasing the adsorption time till 4 h followed by slow uptake rate till 8 h. After 8 h of contact time, the equilibrium was observed and hence, no distinguished adsorption of metal ions was recorded after that time on the adsorbents. This indicated that both the complete saturation of the sorption sites and the quick diffusion of the metal ions onto the adsorbents’ surface had occurred.

#### 3.3.2. Effect of Temperature

**Figure 8:**
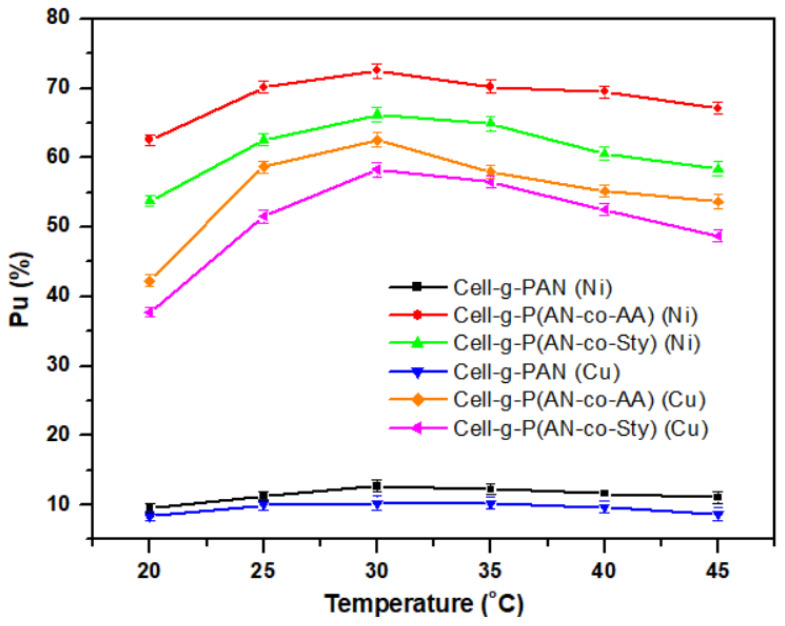
Effect of temperature on the adsorption ratio of different adsorbent cellulose samples at pH = 5.5, time = 8 h, metal ion concentration = 600 mg/L, volume = 50 mL, adsorbent amount = 0.05 g.

Temperature effect on the metal ion adsorption was examined within the range of 20–45 °C (Figure 8). The adsorption efficiencies of Ni(II) and Cu(II) ions increase with increasing medium temperature till 30 °C, then decrease with further increases in temperature. The lower adsorption ratio of metal ions onto the adsorbents may be attributed to the slower diffusion rate of the metal ions and the fast desorption rate compared with the adsorption rate.

#### 3.3.3. Effect of Metal Ion Concentration

**Figure 9:**
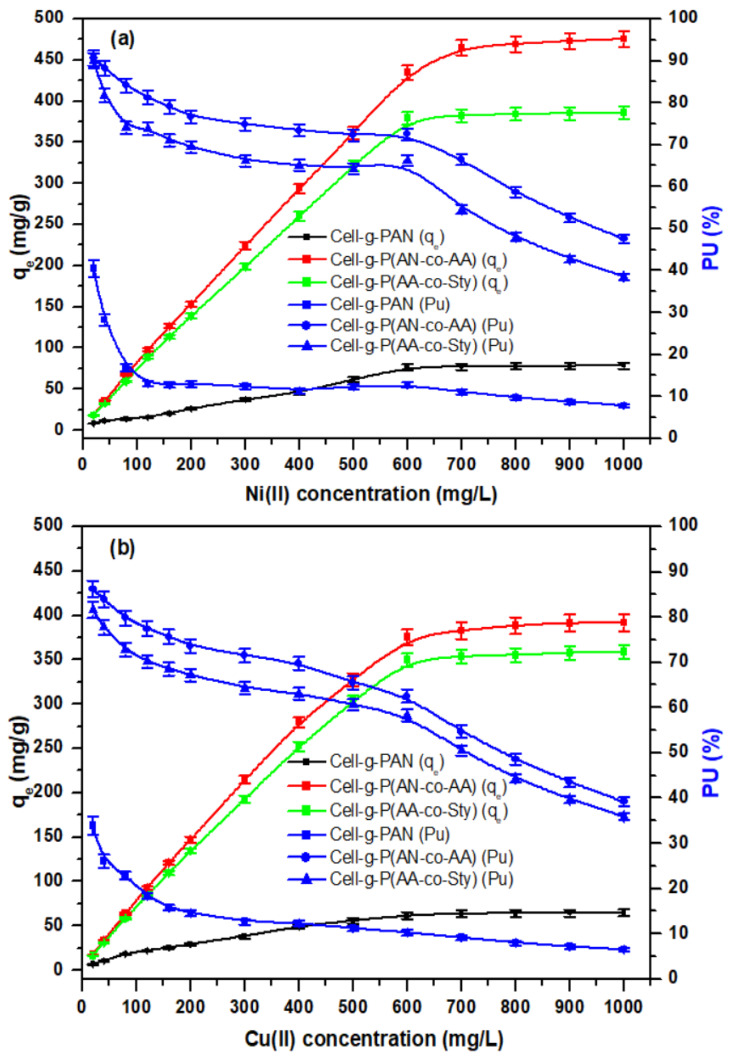
Effect of the metal ion concentration (**a**) Ni(II), (**b**) Cu(II) on the adsorption efficiency of cellulose graft copolymer derivatives at contact time = 8 h., pH = 5.5, T = 30 °C, adsorbent amount = 0.05 g, V = 50 mL.

To study the effect of metal ion concentration, the adsorption rate and percent uptake were recorded with pure cellulose, Cell-g-PAN, Cell-g-P(AN-co-AA), and Cell-g-P(AN-co-Sty) with initial metal ion concentration in the range 20–1000 mg/L at the optimized adsorption conditions of pH 5.5, 8 h contact time, and temperature of 30 °C, as shown in Figure 9a,b. The amount of Ni(II) and Cu(II) ions uptake by Cell-g-PAN and Cell-g-P(AN-co-Sty) was increased with metal ion concentration till 600 mg/L. In the case of Cell-g-P(AN-co-AA), the adsorption efficiency of Ni(II) reached equilibrium at concentration 700 mg/L. Adsorption ratios decrease with metal ion concentration, and the adsorption capacity is constant after 700 mg/L of initial concentration, which indicated that the active sites on graft copolymer becomes lower and there are no sufficient active adsorption sites on the adsorbents to remove higher number of the metal ions. In Figure 9b, cellulose graft copolymer derivatives show the same behavior in the Cu(II) ion adsorption, but lower than adsorption behavior towards Ni(II) ions. Cell-g-P(AN-co-Sty) and Cell-g-P(AN-co-AA) adsorb about 5- and 6-times adsorption amount compared to Cell-g-PAN. The adsorption capacities of Cell-g-PAN for Ni(II) and Cu(II) at the initial metal concentration 600 mg/L were 76.2 mg/g (Pu of 12.7% Pu) and 61.56 mg/g (Pu of 10.26%), respectively. Also, at 600 mg/L initial metal ion concentration, adsorption capacities of Cell-g-P(AN-co-AA) for Ni(II) and Cu(II) were 435.07 mg/g (Pu of 72.51%) and 375.48 mg/g (Pu of 62.58%), respectively. On the other hand, the adsorption efficiencies of Cell-g-P(AN-co-Sty) for Ni(II) and Cu(II) were 379.2 mg/g (Pu of 66.2%) and 349.68 mg/g (Pu of 58.28%), respectively.

From the above results, and by comparing the adsorption efficiency to pure Cell and Cell-g-PAN with adsorption efficiency of Cell-g-P(AN-co-AA) and Cell-g-P(AN-co-Sty), it was observed that excellent adsorbents for metal ions are in the order of: Cell-g-P(AN-co-AA) > Cell-g-P(AN-co-Sty) > Cell-g-PAN > pure-Cell and adsorption efficiency of metal ions is in the order of: Ni(II) > Cu(II) at the optimized adsorption conditions of pH 5.5, 8 h. contact time, and 30 °C temperature. The enhanced metal ion adsorption capacity of the cellulosic material brought about by the incorporation of vinyl monomer can be linked to the presence of the nitrile, phenyl and carboxylic groups on the grafts which provide additional adsorption sites for the metal ions [46]. At comparative manner of graft copolymerization, the adsorption of metal ions from aqueous solution by Cell-g-P(AN-co-AA) is relatively higher than by Cell-g-P(AN-co-Sty) due to the presence of more carboxylic group adsorbing sites.

#### 3.3.4. Adsorption Isotherm Studies

**Figure 10:**
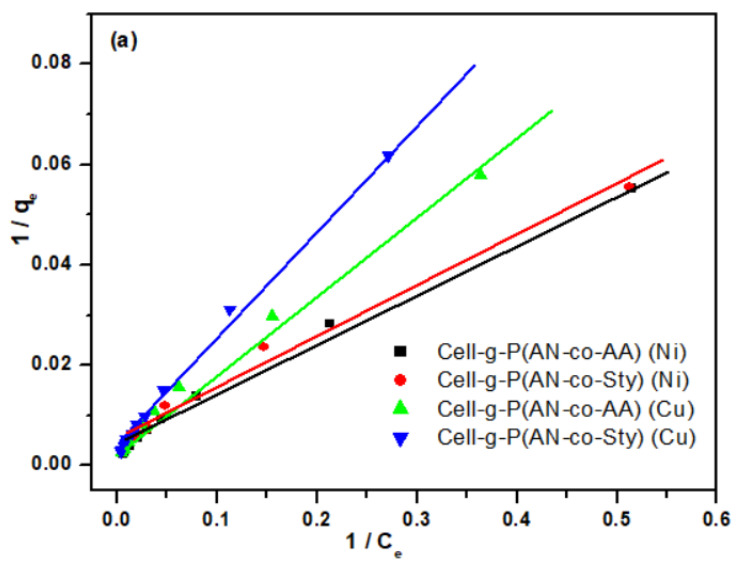

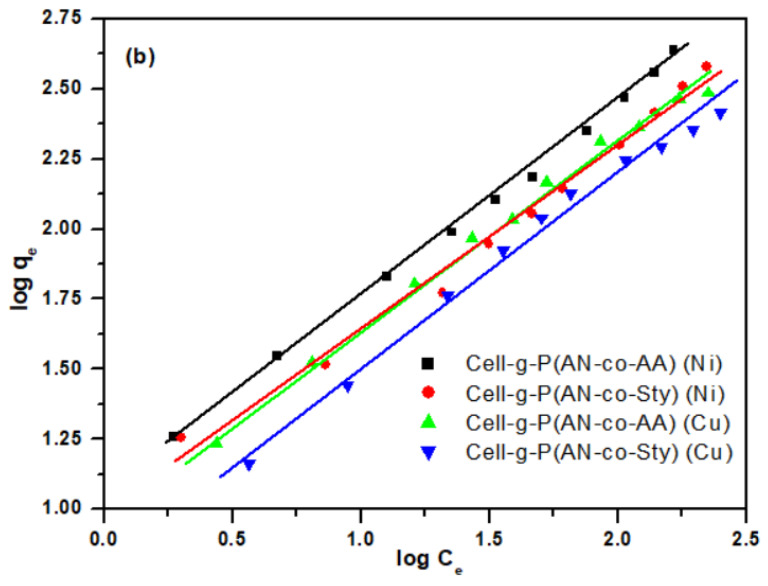
Langmuir (**a**) and Freundlich (**b**) isotherm models for the adsorption of Ni(II) and Cu(II) at contact time = 8 h, pH = 5.5, T = 30 °C, adsorbent amount = 0.05 g, and V = 50 mL.

In order to explain the adsorption behavior and mechanism of Cell-g-P(AN-co-AA) and Cell-g-P(AN-co-Sty) towards Ni(II) and Cu(II) metal ions, the linear forms of Freundlich and Langmuir adsorption isotherm were applied. Their related parameters were determined for Ni(II) and Cu(II) metal ions with concentration range 20–600 mg/L at the optimized adsorption conditions (Figure 10a,b). The Freundlich model was frequently used to characterize the surface’s inhomogeneity. According to the Langmuir model, adsorption takes place at the adsorbent’s outside interface. The homogenous adsorbent surface and equivalent adsorbent sites were postulated by the Langmuir adsorption isotherm model [68]. As a result, the adsorbate monolayer forms on the adsorbent surface. The following equations represent the Freundlich and Langmuir models [69].

(4) $$Freundlich\;model\;\;\;\;\;\;\;\log q_{e}\;\;\;\;=\frac{1}{n}logC_{e}+logK_{F}$$

(5) $$Langmuir\;model\;\;\;\;\;\;\;\;\;\;\;\;\;\frac{1}{q_{e}}=\frac{1}{q_{m}K_{L}}\frac{1}{C_{e}}+\frac{1}{q_{m}}$$

where C_e_ is the Ni(II) and Cu(II) equilibrium concentrations in medium (mg/L), n is the Freundlich exponent interrelated to adsorption power (Heterogeneity factor), K_F_ represents the Freundlich constant (mg/g), q_m_ represents the maximum adsorption efficacy (mg/g), K_L_ is the Langmuir constant (L/mg), and q_e_ is the adsorption capacity (mg/g).

Figure 10a,b and Table 4 represent the fitting curves and fitting parameters for the isotherm models, respectively. The Langmuir adsorption isotherm was found to be the most appropriate model based on correlation coefficient values (R^2^), since its R^2^ values are higher than those of the Freundlich model. This implies that the metal ions chemisorb in a monolayer onto the grafted copolymer surface. The following formula [70] provides the properties and feasibility of the Langmuir model according to the dimensional separation factor (R_L_):

(6) $$R_{L}=\frac{1}{K_{L}C_{i}+1}$$

where C_i_ is the initial concentration of Ni(II) and Cu(II). The R_L_ values verify if the adsorption is irreversible (R_L_ = 0), linear (R_L_ = 1), unfavorable (R_L_ > 1), or favorable (0 < R_L_ < 1). The values of R_L_ for Cell-g-P(AN-co-AA) and Cell-g-P(AN-co-Sty) were less than 1, which indicate suitable adsorption isotherm type of Ni(II) and Cu(II) metal ions onto the Cell-g-P(AN-co-AA) and Cell-g-P(AN-co-Sty) surfaces in the investigated concentration range. The n value, which reflects the adsorption route’s favorability, varies with sorbent heterogeneity. Moreover, the value of R_L_ < 1 and n > 1 both indicated that graft copolymer absorbents had favorable adsorption behavior.

#### 3.3.5. Comparative Study

Comparative adsorption capacities of Cell-g-P(AN-co-AA) and Cell-g-P(AN-co-Sty) sorbents for Ni(II) and Cu(II) removal with other reported similar sorbents are listed in Table 5. Compared to previously published data, the sorption efficiencies of the Cell-g-P(AN-co-AA) and Cell-g-P(AN-co-Sty), which were synthesized by the simple graft copolymerization method used KMnO_4_/citric acid redox initiator, are significantly higher. For example, Cell-g-AASO3H-co-AAc has qmax of 112.74 and 109.77 mg/g for Ni(II) and Cu(II) metal ions [32] as listed in Table 5.

## 4. Conclusions

New biodegradable Cell-g-P(AN-co-AA) and Cell-g-P(AN-co-Sty) graft copolymers were prepared by KMnO_4_/citric acid redox initiator and applied to absorb the heavy metal ions. The maximum GY (%) was investigated at binary monomer mixture ratio of 60:40 of the total volume of the monomer examined. The adsorption behaviors of cell-g-P(AN-co-AA) and Cell-g-P(AN-co-Sty) were investigated for the removal of Ni(II) and Cu(II) from aqueous medium. The maximum adsorption capacities of Ni(II) and Cu(II) metal ions, recorded at the optimal adsorption efficient conditions of 8 h contact time, pH 5.5, and 30 °C temperature, were 435.07 mg/g and 379.2 mg/g, respectively for Cell-g-P(AN-co-AA) and 375.48 mg/g and 349.68 mg/g, respectively for Cell-g-P(AN-co-Sty), which are higher than those for Cell-g-PAN by five or six times. The adsorption mechanism follows the Langmuir isothermal model, where metal ions interact chemically with grafted cellulose in monomer mixtures. In conclusion, Cell-g-P(AN-co-AA) and Cell-g-P(AN-co-Sty) were found to have an inexpensive and significant heavy metal adsorption capacity. Remarkably, the graft copolymerization of cellulose fiber with binary monomer mixtures for heavy metal wastewater removal promotes both environmental and economic sustainability.

## Figures and Tables

**Table 1:** Effect of different monomer concentration and binary monomer mixture ratio of (10% v monomer conc.) on the percent graft yield, time = 60 min, [KMnO_4_] = 0.05 mol/L, [citric acid] = 0.02 mol/L, T = 70 °C.

Monomer (V%)	Percent Graft Yield (GY%)			Monomer Ratio (10%) (AN: Vinyl Monomer)	Percent Graft Yield (GY%)	
	AN	AA	Sty		AN:AA	AN:Sty
2	7.8	00.0	00.0	100:00	69.3	69.3
4	19.8	00.0	00.0	80:20	54.9	43.4
6	49.1	00.0	00.0	60:40	71.3	79.5
8	68.4	00.0	00.0	40:60	48	23.2
10	69.3	3.3	00.0	20:80	13.2	4.2

**Table 2:** Thermal analysis data the grafted cellulose with different monomers and different monomers mixtures.

Sample	(IDT-FDT °C) Mass Loss of Stages (%)				Residue (%) at 530 °C
	First	Second	Third	Fourth	
UN-C	28–115 °C 6%	270–382 °C 75.5%	--	--	2.00%
Cell-g-PAN	28–103 °C 4%	250–300 °C 10.3%	300–384 °C 37.2%	--	33%
Cell-g-P(AN-co-AA)	28–100 °C 4.5%	235–300 °C 4.2%	300–370 °C 49.9%	370–465 °C 15.13%	25%
Cell-g-P(AN-co-Sty)	28–150 °C 3.4%	260–320 °C 7.7%	320–380 °C 33.3%	390–434 °C 39.11%	9%

**Table 3:** Crystallinity degree (Cr%) of grafted cellulose with different monomers and different monomers mixtures, monomer concentration = 10 v%, Cr% of untreated cellulose = 78.6%.

Monomers Ratio (AN Monomer) Total Conc. = 10 v%	Degree of Crystallinity (Cr%)		Monomer Concentration (v%)	Degree of Crystallinity (Cr %)
	AN:AA	AN:Sty		AN
100:00	46.2	46.2	10	46.2
80:20	52.6	42.9	8	47.3
60:40	53.3	41.7	6	50.0
40:60	55.2	44.7	4	53.0
20:80	57.7	55.6	2	57.1
00:100	69.5	--		

**Table 4:** Adsorption isotherm parameters for Ni(II) and Cu(II) adsorption onto grafted cellulose with binary monomer mixtures.

Isotherm	Constants	Cell-g-P(AN-co-AA)		Cell-g-P(AN-co-Sty)	
		Ni(II)	Cu(II)	Ni(II)	Cu(II)
Langmuir	K_L_ (L/mg)	0.0018	0.0016	0.0017	0.0013
	q_m_ (mg/g)	446.43	384.62	387.59	362.32
	R^2^	0.9989	0.9997	0.9988	0.9991
	R_L_	0.7976	0.5142	0.4905	0.5709
Freundlich	K_F_ (L/mg)	11.5659	9.4146	9.7029	6.6039
	n	1.4432	1.5051	1.5058	1.4535
	R^2^	0.9981	0.9941	0.9962	0.9942

**Table 5:** Comparison of Adsorption Capacity for Ni(II) and Cu(II) Removal for different Adsorbents.

Adsorbent	q_max_ (mg/g)		C_i_ (mg/L)	pH	T (°C)	Time h	Ref.
	Ni (II)	Cu(II)					
Cell-g-AASO3H-co-AAc	112.74	109.77	200	6	30	2	[32]
CC-g-(AA-co-AM)	--	157.51	600	5	27	0.5	[35]
CE–PAANa	--	106.3	600	5		24	[37]
Cell-g-NIPAM-co-AAc	79.78	84.67	200	5	30	6	[55]
Cell-g-NIPAM-co-GMA	74.68	82.92	200	6 (Ni) 5(Cu)	30	6	[56]
Cell-g-HEMA-co-GMA	83.80	71.40	200	6	30	6	[57]
Cell-g-HEMA-co-AAc	85.32	84.82	200	5 (Ni) 6(Cu)	30	6	[59]
Cell-g-P(AN-co-AA)	153.21	147.2	200	5.5	30	8	Current work
Cell-g-P(AN-co-Sty)	139	134.42	200	5.5	30	8	Current work
Cell-g-P(AN-co-AA)	435.07	375.48	600	5.5	30	8	Current work
Cell-g-P(AN-co-Sty)	379.2	349.68	600	5.5	30	8	Current work

## Data Availability

All data analyzed during this study are included in this published article.

## Supplementary Materials

The following supporting information can be downloaded at: https://www.mdpi.com/article/10.3390/polym16030445/s1.
