# Supplementary material for: Synthesis, Characterization, and Evaluation of the Adsorption Behavior of Cellulose-Graft-Poly(Acrylonitrile-co-Acrylic Acid) and Cellulose-Graft-Poly(Acrylonitrile-co-Styrene) towards Ni(II) and Cu(II) Heavy Metals
Source: Polymers (Basel). 2024 Feb 5;16(3):445. doi: 10.3390/polym16030445 (PMC10857649; doi:10.3390/polym16030445)
Supplement: Supplementary file 1 [file polymers-16-00445-s001.zip › polymers-2798046-supplementary.pdf]

Supplementary figures

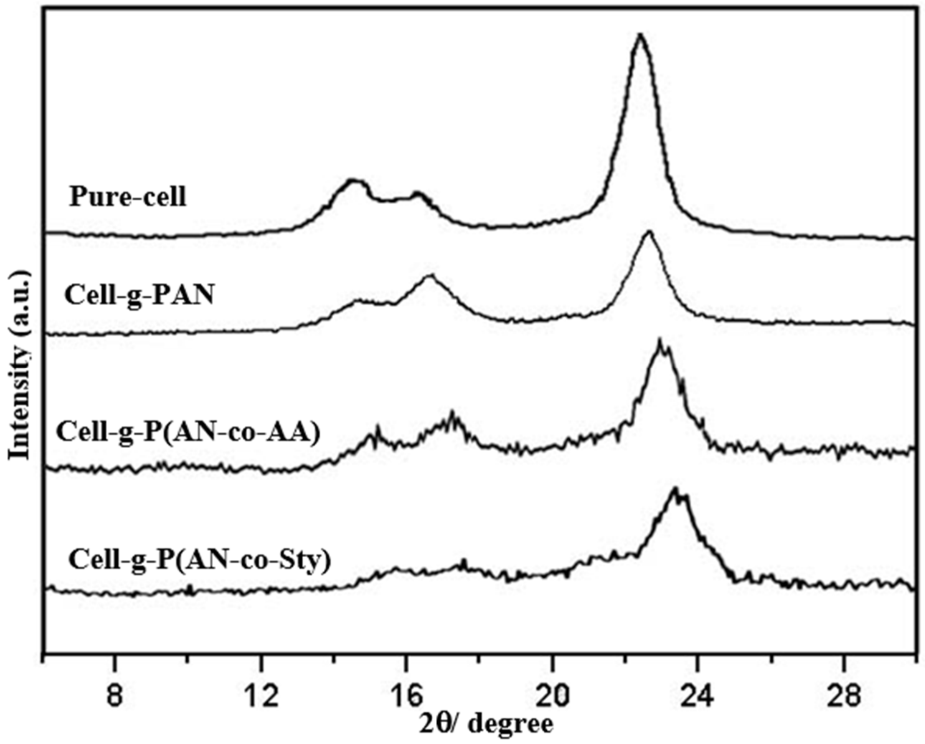

Figure S1. a: X-ray diffraction of pure cellulose and grafted cellulose with monomer mixture of 80:20 ratio of AN/vinyl monomer.

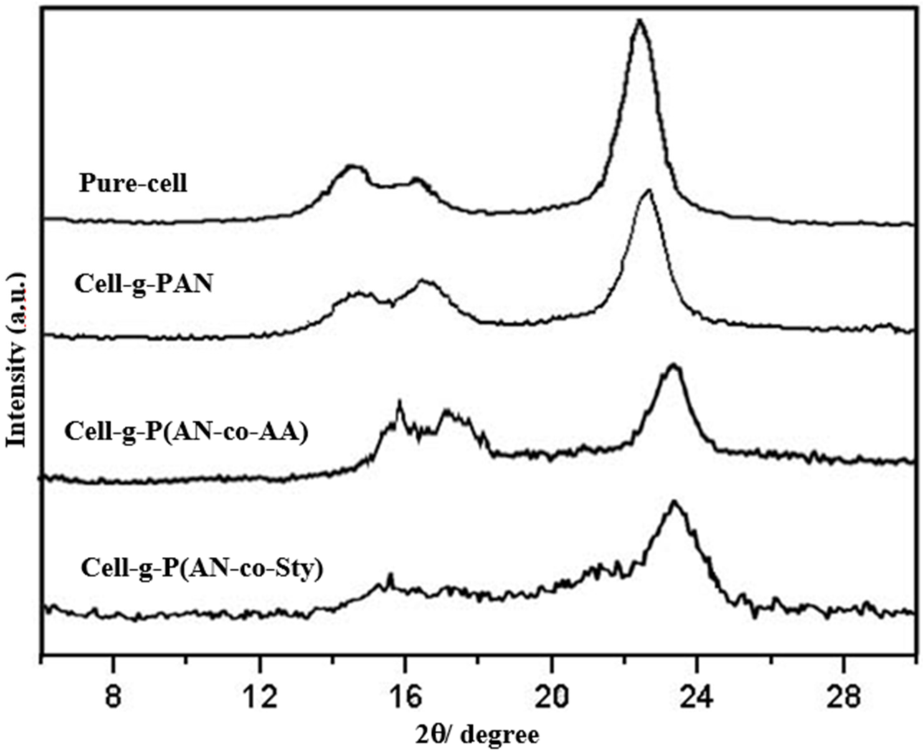

Figure S1. b X-ray diffraction of pure cellulose and grafted cellulose with monomer mixture of 40:60 ratio of AN/vinyl monomer.

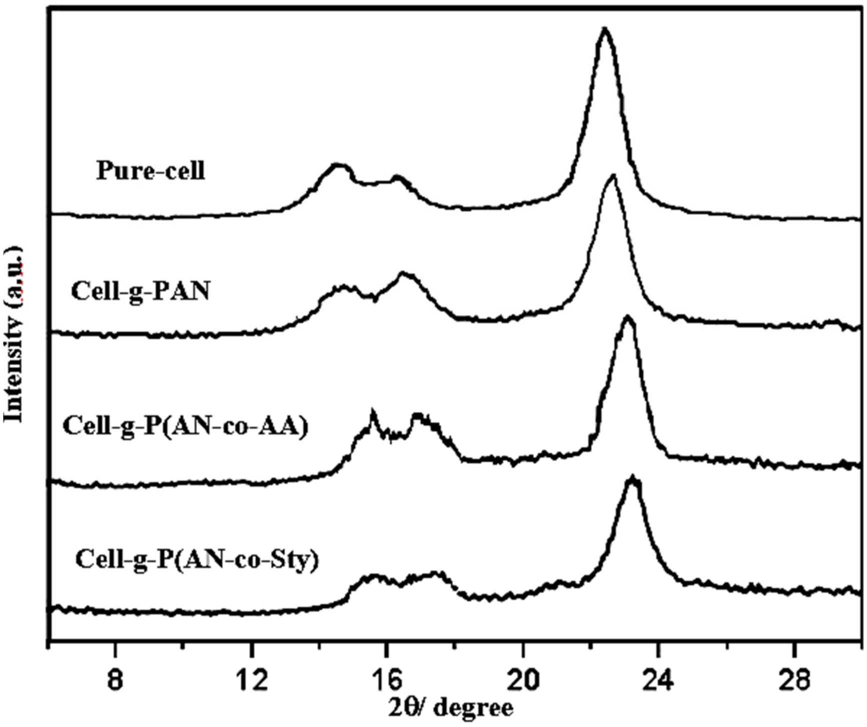

Figure S1. c: X-ray diffraction of pure cellulose and the grafted cellulose with monomer mixture of 20:80 ratio of AN/vinyl monomer.

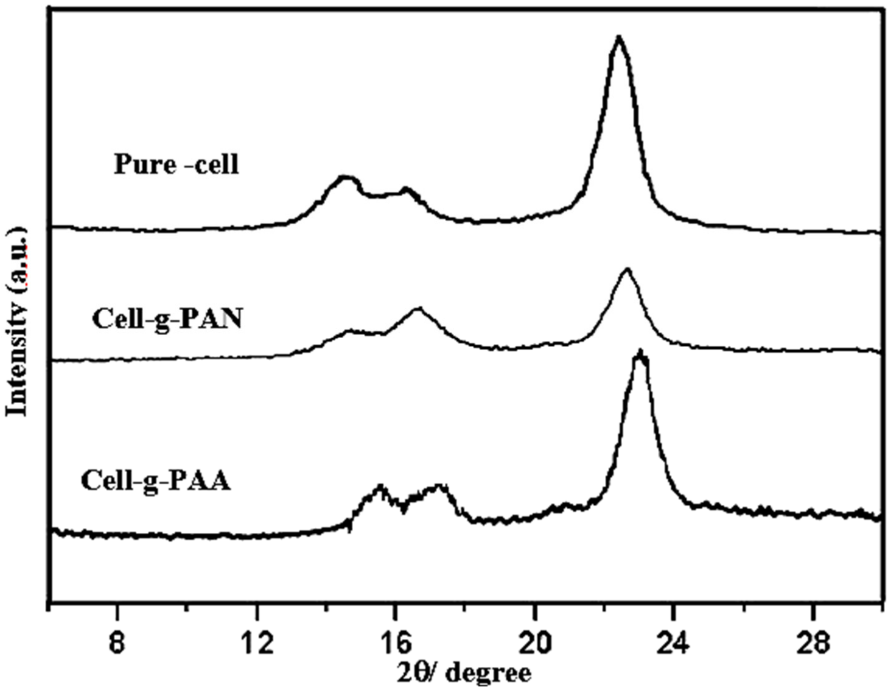

Figure S1. d: X-ray diffraction of pure cellulose and the grafted cellulose with different monomer, monomer Concentration = 10 v%.
